# Comparison of Clinical Presentation and Vaccine Effectiveness Among Omicron and Non-omicron SARS Coronavirus-2 Patients

**DOI:** 10.7759/cureus.32354

**Published:** 2022-12-09

**Authors:** Monalisa Mohanty, Baijayantimala Mishra, Arvind K Singh, Prasanta R Mohapatra, Kavita Gupta, Binod K Patro, Dinesh P Sahu, Punyatoya Kar, Prashanth Purushotham, Swarnatrisha Saha, Sivasankar Das, Prabhudutta Mamidi, Sailendra Panda, Madhab Charan Mandal, Sourin Bhuniya

**Affiliations:** 1 Department of Microbiology, All India Institute of Medical Sciences, Bhubaneswar, Bhubaneswar, IND; 2 Department of Community Medicine and Family Medicine, All India Institute of Medical Sciences, Bhubaneswar, Bhubaneswar, IND; 3 Department of Pulmonary Medicine and Critical Care, All India Institute of Medical Sciences, Bhubaneswar, Bhubaneswar, IND

**Keywords:** sars-cov-2 (severe acute respiratory syndrome coronavirus -2), non-omicron, covid-19 vaccines, omicron, covid-19

## Abstract

Introduction

The rapidly mutating Omicron SARS-CoV-2 variant has replaced the previous dominant SARS-CoV-2 variants like alpha, and delta resulting in the amplification of coronavirus disease 2019 (COVID-19) cases. The present study was conducted to compare the clinical profile and vaccination status in patients infected with Omicron and non-Omicron SARS-CoV-2 variants.

Methods

All patients who tested positive for coronavirus disease 2019 (COVID-19) during the study period (January 2022 to February 2022) were further tested for detection of SARS-CoV-2 Omicron variant by using Omisure kit (TATA MD CHECK RT-PCR, TATA MEDICAL AND DIAGNOSTICS LIMITED, Tamil Nadu, INDIA). Clinico-demographic factors and vaccination status were compared between both Omicron and non-Omicron groups.

Results

A total of 1,722 patients who tested positive for COVID-19 were included in the study, of which 656 (38.1%) were Omicron and 1,066 (61.9%) were non-Omicron SARS-CoV-2 variants. Blood group and vaccination status were the major predictors for Omicron. The proportion of male patients was 58.4% in the Omicron group and 57.9% in the non-Omicron group. Maximum cases (86.2%) belonged to >18-60 years age group, 7.3% to >60 years age group, and least to 0-18 years (6.5%). The average age of the study participants was 35.4 ± 14.5 years. Vaccinated participants had less chance of having Omicron than the unvaccinated participants (p-value - 0.003). Fever and loss of smell were found to be significantly associated with the non-Omicron SARS-CoV-2 variant. (p-value < 0.05).

Conclusion

The present study reflects that the clinical course of the disease is milder in Omicron as compared to the non-Omicron variant. However rapid rise in cases can badly affect the healthcare system demanding good preparedness to tackle all the predicaments. Good Vaccination coverage should be of utmost priority irrespective of the variant type.

## Introduction

The globe has witnessed massive morbidity and mortality along with severe socioeconomic catastrophes owing to the ongoing pandemic due to the coronavirus disease 2019 (COVID‐19) caused by the novel coronavirus SARS-CoV-2 [1]. The continued emergence of new variants worsens the scenario in terms of the increasing number of human sufferings, vaccine ineffectiveness, and economic burden to society in every continent [1-3]. The emergence of the highly mutated SARS- CoV‐2 variant (B.1.1.529) “Omicron,” replaced the Delta variant in many countries resulting in an upsurge of COVID‐19 cases [3,4]. The first two cases of the Omicron SARS-CoV-2 variant in India were noted on December 2, 2021 and, subsequently, the upsurge of COVID-19 cases occurred since the last week of December 2021 [5,6].

There are over 50 mutations in the Omicron SARS-CoV-2 variant, including more than 32 mutations in the spike protein and more than 15 mutations in the receptor-binding protein [7]. Studies have shown a higher transmission rate of the Omicron SARS-CoV-2 variant over the Delta variant resulting in the latest escalation of COVID-19 cases across countries [8]. However, most of the studies have reported that Omicron SARS-CoV-2 variant causes infections of mild severity as compared to the Delta variant [9]. But the reinfection rate is five-fold higher in the Omicron SARS-CoV-2 variant when compared to the previous dominant Delta variant [9]. A significant drop in the effectiveness of the vaccines against symptomatic infection has also been documented [10]. In different studies, it is observed that there is a remarkable reduction in the neutralization rate against the Omicron SARS-CoV-2 variant both in convalescent sera as well as with sera of vaccinated individuals [11-13]. All these findings allude to the immune evasion property of the Omicron SARS-CoV-2 variant. Sequencing is being done to identify the different variants including Omicron of SARS-CoV-2, correctly. Apart from that, S gene target failure (SGTF) can also be considered a proxy marker to screen for this variant [4].

As per the data from June to July 2021, the seroprevalence of IgG antibodies against SARS-CoV-2 in India was 67.6% [14]. In India, as of January 24, 2022, > 84 million people have received at least one dose, and 60 million have received two doses [15]. India is a country with more than 135 crore population; therefore, the data regarding the Omicron SARS-CoV-2 variant-associated health emergencies such as the use of oxygen, mechanical ventilation, other complications, and the number of deaths is crucial for the smooth management of Omicron cases. In this vein, the present study compared the clinical spectrum, vaccination, and reinfection status in populations infected with Omicron and non-Omicron SARS-CoV-2 variants.

## Materials and methods

This retrospective study was carried out in a tertiary care hospital in Eastern India after getting institutional ethics committee approval (Ref Number: T/IM-NF/Micro/22/03) on all the samples received for COVID-19 RT PCR testing from January 1, 2022 to February 7, 2022. A total of 12,977 were tested for COVID-19 RT PCR. 2,893 were detected as COVID-19 PCR positive and were further tested for the presence of the Omicron SARS-CoV-2 variant and grouped as Omicron positive and negative. Out of 2,893, 1,171 participants refused to participate in the study. Hence, 1,722 participants were included in the present study.

Microbiological testing for COVID-19 and Omicron SARS-CoV-2 variant

One nasopharyngeal swab was collected per individual according to the Indian Council of Medical Research (ICMR) guidelines for COVID-19 RT-PCR testing and sent to the microbiology lab in VTM, maintaining the cold chain [10]. The nucleic acid extraction was performed in an automated platform (King Fischer flex, Thermo Fisher SCIENTIFIC), and RT-PCR testing was done (BIO RAD CFX96) according to the manufacturer’s instructions. All the 2,893 samples which came positive for COVID-19 were further subjected to Omicron SARS-CoV-2 variant testing using the OmiSure kit (TATA MD CHECK RT-PCR, TATA MEDICAL AND DIAGNOSTICS LIMITED, Tamil Nadu, INDIA) by real-time PCR. Based on the Omisure RT-PCR results, patients with the absence of S gene (i.e., SGTF) with or without the presence of mutated S gene (S gene MA or SGMA) were considered to be Omicron SARS-CoV-2 variant positive. The rest cases with the presence of the S gene belonged to the non-Omicron SARS-CoV-2 category.

All the COVID-19 RT PCR tested positive patients between January 1, 2022 and February 7, 2022 were enrolled consecutively in the excel sheet. A set of questionnaires was prepared, and the participants of both the study groups (Omicron positive and non-Omicron SARS-CoV-2 patients) were contacted telephonically. Information on demographic data, clinical data, and vaccine status from both groups were collected and compared among patients who gave consent.

Statistical analysis

All the data already available in the laboratory were collated in an excel sheet, and statistical analysis was done using STATA by using SPSS version 22. The outcome variable in the study was the type of strain (i.e., Omicron [Group 1] or non-Omicron [Group 2]). The clinical characteristics of patients in Group 1 and Group 2 were compared using appropriate statistical tests (Chi-square categorical variables). A P-value less than 0.05 was considered significant. The proportion of reinfection and vaccination in the two groups was also compared using the same statistical tests. Multivariate logistic regression was performed with the type of variant as the outcome variable and sociodemographic variable, vaccination status, and reinfection as a predictor variable.

## Results

**Figure 1:**
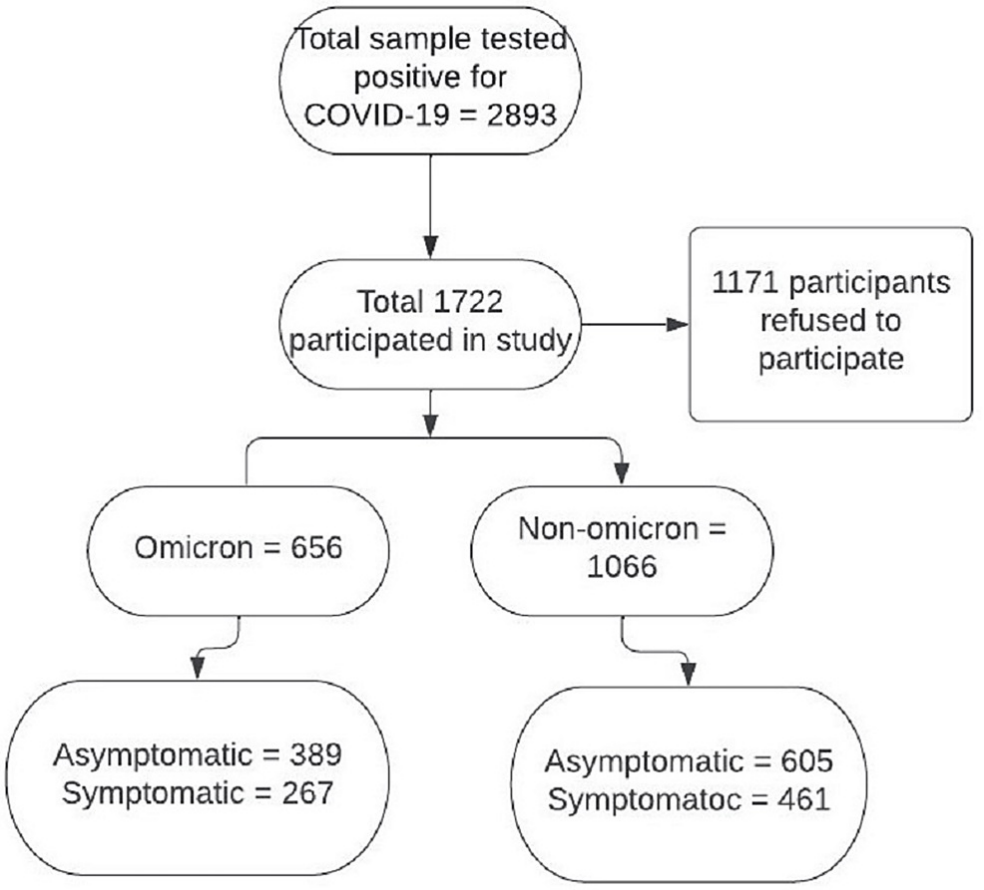
Distribution of cases in Omicron and non-Omicron groups

**Figure 2:**
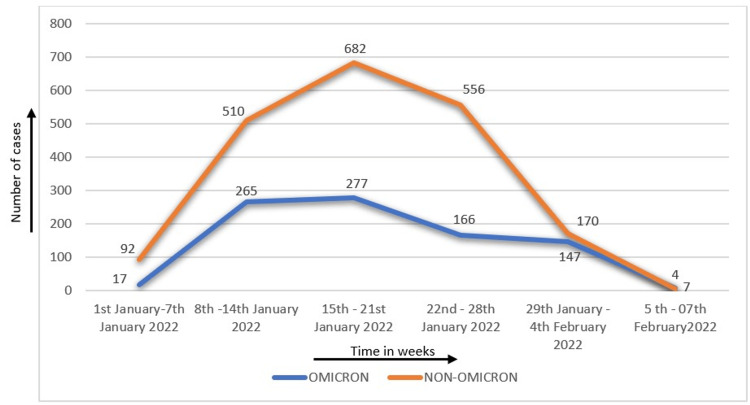
Weekly distribution of Omicron and non-Omicron cases (N=1,722)

A total of 12,977 patients were tested for COVID-19 RT PCR during the defined study period, of which 2,893 (22.3%) samples were positive for SARS-CoV-2 RTPCR. Of these 2,893 COVID-19-positive samples, 879 were Omicron SARS-CoV-2 variant positive (30.4%), and 2,014 (69.6%) were non-Omicron SARS-CoV-2 variant. Of the 2,893 COVID-19-positive patients, only 1,722 participants were included in the study, of which 656 were Omicron SARS-CoV-2 variant positive and the rest 1,066 were negative for Omicron SARS-CoV-2 variant as depicted in Figure 1. On analyzing the trend of omicron SARS-CoV-2 variant positivity, it was observed that the incidence of Omicron SARS-CoV-2 variant positive cases increased gradually from two positives (3.3%) during the beginning of January 2022 to a maximum of 277 positive cases amounting to 45.5% of total SARS-CoV-2 positive cases, which occurred during the third week of January shown in Figure 2.

Both in Omicron and non-Omicron groups, the proportion of male patients was almost approximately 60% (58.4% in the Omicron group and 57.9% in the non-Omicron group). The study population was categorized into three age groups: 0-18 years, >18-60 years, and >60 years. In both Omicron SARS-CoV-2 variant positive and non-Omicron groups, 86.2% belonged to >18-60 years age group, 7.3% belonged to >60 years age group and 6.5% in 0-18 years age group. Average age of the study participants was 35.4 ± 14.5 years.

The proportion of symptomatic patients among the Omicron SARS-CoV-2 variant positive group was lower, i.e., 40.7% as compared to non-Omicron cases 43.2% (Table 1). In all the age groups, >85% of individuals were vaccinated and >60% patients among them vaccinated with two dosages of the vaccine. Reinfection rate was found to be higher in symptomatic and asymptomatic Omicron SARS-CoV-2 variant positive patients (13.1% and 16.5%, respectively) than non-Omicron SARS-CoV-2 variant patients, i.e., 11.7% and 13.9%, respectively (Table 1).

**Table 1:** Demographic and clinical profile of Omicron and non-Omicron patients (N=1,722)

		Omicron N (%)		Non-Omicron	
Variables	Categories	Symptomatic n=267	Asymptomatic n=389	Symptomatic n=461	Asymptomatic n=605
Cause of testing in asymptomatic cases	Symptomatic	267 (100.0)	0 (0.0)	461 (100.0)	0 (0.0)
	Cancer chemotherapy or before surgery	0 (0.0)	288 (74.0)	0 (0.0)	279 (46.1)
	Self tested	0 (0.0)	48 (12.3)	0 (0.0)	187 (30.9)
	Patient attendant	0 (0.0)	25 (6.4)	0 (0.0)	87 (14.4)
	Travel	0 (0.0)	6 (1.5)	0 (0.0)	17 (2.8)
	Job interview	0 (0.0)	15 (3.8)	0 (0.0)	13 (2.2)
	Contact with COVID-19 positive case	0 (0.0)	7 (1.8)	0 (0.0)	22 (3.6)
Blood group	A-ve	2 (0.7)	12 (3.1)	2 (0.4)	9 (1.5)
	A+ve	35 (13.1)	64 (16.5)	93 (20.1)	98 (16.2)
	AB-ve	1 (0.3)	2 (0.5)	2 (0.4)	1 (0.1)
	AB+ve	15 (5.6)	18 (4.6)	40 (8.7)	21 (3.4)
	B-ve	5 (1.9)	11 (2.8)	5 (1.1)	7 (1.1)
	B+ve	85 (31.8)	119 (30.5)	122 (26.5)	198 (32.7)
	O-ve	4 (1.5)	6 (1.5)	8 (1.7)	3 (0.5)
	O+ve	119 (44.6)	148 (38.0)	169 (36.7)	243 (40.1)
	Not available	3 (1.1)	7 (1.8)	20 (4.3)	25 (4.1)
Reinfection	No	232 (86.9)	325 (83.5)	407 (88.3)	521 (86.1)
	Yes	35 (13.1)	64 (16.5)	54 (11.7)	84 (13.9)
Hospitalization	No	256 (95.9)	377 (96.9)	416 (90.2)	574 (94.9)
	Yes	11 (4.1)	12 (3.1)	45 (9.8)	31 (5.1)
Vaccination status	Unvaccinated	28 (10.5)	21 (5.4)	31 (6.7)	10 (1.6)
	Vaccinated	234 (87.6)	357 (91.7)	414 (89.8)	571 (94.4)
	Data not available	5 (1.9)	11 (2.8)	16 (3.5)	24 (4.0)
Type of vaccination	Fully vaccinated (two doses)	167 (62.5)	268 (68.9)	304 (65.9)	447 (73.9)
	Three doses	9 (3.4)	14 (3.6)	42 (9.1)	28 (4.6)
	Two doses (<14 days)	16 (5.6)	9 (2.3)	31 (6.7)	28 (4.6)
	Partial vaccinated	12 (4.5)	19 (4.9)	29 (6.2)	31 (5.1)
	One dose (<21 days)	30 (11.2)	47 (12.1)	8 (1.7)	37 (6.1)

In both the study groups, fever was the predominant symptom (62% in Omicron and 83.3% in non-Omicron cases) followed by cold, cough, and body ache. The presence of fever, loss of smell and hospitalization were significantly associated with the non-Omicron variant as shown in Table 2.

**Table 2:** Comparison of clinical profile among symptomatic Omicron positive and symptomatic non-Omicron patients (N=728)

Characteristics	Categories	Symptomatic Omicron (n = 267) N (%)	Symptomatic non-Omicron (n = 461) N (%)	P-value
Symptoms	Fever	168 (62.9)	384 (83.3)	<0.001
	Cold	77 (28.8)	157 (34.1)	0.146
	Cough	50 (18.7)	110 (23.8)	0.106
	Sore throat	18 (6.7)	33 (7.1)	0.831
	Shortness of breath	3 (1.1)	2 (0.4)	0.277
	Body ache	51 (19.1)	105 (22.8)	0.244
	Head ache	27 (10.1)	39 (8.4)	0.448
	Loss of taste	1 (0.4)	7 (1.5)	0.153
	Loss of smell	0 (0.0)	15 (3.2)	0.010
	Diarrhea	3 (1.1)	1 (0.2)	0.110
	Vomiting	1 (0.4)	4 (0.8)	0.437
	Death	2 (0.7)	3 (0.6)	0.877
Reinfection	Yes	35 (13.1)	54 (11.7)	0.580
	No	232 (86.9)	407 (88.3)	
Hospitalization	Yes	11 (4.1)	45 (9.8)	0.006
	No	256 (95.9)	416 (90.2)	

Blood group and the vaccination status were the major predictors of the Omicron and non-Omicron variants of SARS-CoV-2 variants. The patients with Omicron variants had 1.092 times higher chance of hospitalization than that of the non-Omicron patients. Vaccinated participants had less chance of having the Omicron SARS-CoV-2 variant than the unvaccinated participants. (Table 3). A total of five deaths were reported during the study period, of which two had Omicron SARS-CoV-2 variant and three were non-Omicron SARS-CoV-2 variant. Of the five patients who died, four were unvaccinated.

**Table 3:** Comparison of Omicron and non-Omicron cases (N=1,722)

Variables	Categories	Omicron	Non-Omicron	Total	P-value	OR (95% CI)
Gender	Male	387 (38.3)	624 (61.7)	1011 (100.0)	0.852	Reference
	Female	269 (37.8)	442 (62.2)	711 (100.0)		0.975 (0.798-1.191)
Age group	0-17 years	37 (33.9)	72 (66.1)	109 (100.0)	0.613	Reference
	18-59 years	567 (38.3)	915 (61.7)	1482 (100.0)		1.201 (0.791-1.824)
	≥60 years	52 (39.7)	79 (60.3)	131 (100.0)		1.302 (0.759-2.231)
Blood group	A-ve	14 (56.0)	11 (44.0)	25 (100.0)	0.007	Reference
	A+ve	99 (34.1)	191 (65.9)	290 (100.0)		0.407 (0.178-0.930)
	AB-ve	3 (50.0)	3 (50.0)	6 (100.0)		0.786 (0.132-4.680)
	AB+ve	33 (35.1)	61 (64.9)	94 (100.0)		0.425 (0.173-1.041)
	B-ve	16 (57.1)	12 (42.9)	28 (100.0)		1.048 (0.353-3.110)
	B+ve	204 (38.9)	320 (61.1)	524 (100.0)		0.501 (0.223-1.125)
	O-ve	10 (47.6)	11 (52.4)	21 (100.0)		0.714 (0.223-2.290)
	O+ve	267 (39.3)	412 (60.6)	679 (100.0)		0.509 (0.228-1.138)
Symptoms	Asymptomatic	389 (39.1)	605 (60.9)	994 (100.0)	0.299	Reference
	Symptomatic	267 (36.7)	461 (63.3)	728 (100.0)		0.921 (0.754-1.124)
Hospitalized	Yes	23 (23.2)	76 (76.8)	99 (100.0)	0.002	1.092 (1.172-3.088)
	No	633 (39.0)	990 (61.0)	1623 (100.0)		Reference
Reinfection	Yes	99 (41.8)	138 (58.2)	237 (100.0)	0.289	1.164 (0.879-1.541)
	No	557 (37.5)	928 (62.5)	1485 (100.0)		Reference
Vaccination status	Unvaccinated	49 (54.4)	41 (45.6)	90 (100.0)	0.003	Reference
	Vaccinated	591 (38.2)	955 (61.8)	1546 (100.0)		0.518 (0.337-0.793)

## Discussion

The present study has attempted to compare and correlate the clinical profile, vaccination status, and rate of reinfection and hospitalization between Omicron and non-Omicron patients. The quick escalation of Omicron SARS-CoV-2 variant cases within two weeks spans as observed in this study is consistent with the fact of high transmissibility property of the Omicron SARS-CoV-2 variant; as a consequence of being a rapidly evolving, mutation-prone RNA virus with a striking power of reproduction (3.6-4.2 times) than the previous variant of concern (VOC), i.e., delta variant [8,16]. Additionally, the horde of >50 mutations (32 in spike protein and 17 in receptor binding protein) atone for the exalted binding capacity of the Omicron SARS-CoV-2 variant to angiotensin-converting enzyme 2 receptor (ACE2-R) (e.g., N501Y, T478K) and/or increased splitting of host furin at the spike protein (e.g., P681H, N679K) expedites the infectivity and transmissibility of this variant [7,16].

Though most cases were asymptomatic, the hospitalization rate was more frequent in the non-Omicron group, but the reinfection rate was higher among the Omicron SARS-CoV-2 variant positive group; albeit, in all subgroups, vaccine coverage was more than 85%, with >60% of cases received two dosages of the vaccine. Both the groups had a similar clinical spectrum, but the course of illness was milder among Omicron SARS-CoV-2 variant positive cases, reflecting its enervated pathogenicity in contrast to previous VOCs and simultaneously emphasizing the significance of vaccination in lessening the severity and fatal outcome [16,17].

Fever is the most common symptom affecting 83.3% of non-Omicron and 62% of Omicron patients, followed by cold, cough, and body aches, per the findings of other studies [18]. But the loss of smell and taste sensation was more prevalent in non-Omicron individuals. Both groups found shortness of breath in a few symptomatic cases. In Omicron symptomatic cases, it might be attributed to the decreased ability of the Omicron SARS-CoV-2 variant to replicate in lungs as seen in different animal models [19], and in the non-Omicron SARS-CoV-2 variant group might be due to non-inclusion of all cases. According to the observations of previous studies ‘O’, the blood group was found to be resistant to different variants of SARS-CoV-2 infection [20,21], but in our study, the O-blood group type was the most commonly affected type. This might be because the previous studies took admitted patients into account, while the present study included hospitalized and non-hospitalized patients; however, the overall hospitalization rate in the current study is lower than in the previous study [17]; the difference may be due to the difference in the initial State policy of mandatory admission of all the suspected Omicron cases and close clinical monitoring of the patients [17].

The reinfection rate in all the subgroups in the present study was more than 10% which is slightly higher than the observations by other studies with reinfection rates of 6.2%, and 5.9%, respectively [17,22]. The higher reinfection rate could correspond to the variation in the vaccine coverage rate in different regions and the development of immunity among individuals because of earlier COVID-19 infection [22]. Moreover, the possibility of the immune evasion capability of different SARS-CoV-2 variants cannot be ignored, as a mutation in the E484A amino acid in the spike protein can result in the depletion of neutralizing capability of the host, which unlocks the door of immune escape for the virus [16]. In the present study, death was reported in five individuals (two Omicron SARS-CoV-2 variant positive and three non-Omicron SARS-CoV-2 variant cases), and four of the five deceased persons were not vaccinated for COVID-19. The lower mortality rate is analogous to other studies pertaining to the milder course of the Omicron SARS-CoV-2 variant than other non-Omicron SARS-CoV-2 variants [17]. Though the number is less, the death of four unvaccinated patients in the present study again emphasized the importance and protectiveness of vaccination in COVID-19 disease regardless of different variants. The incidence of the Omicron SARS-CoV-2 variant was less reported in the vaccinated participants. This suggested that vaccines were protective against the Omicron SARS-CoV-2 variant also.

Our study had several limitations. All the patients in both the Omicron SARS-CoV-2 variant and non-Omicron SARS-CoV-2 variant groups could not be included either due to denial to give consent or because of wrong contact information. The inclusion criteria of SGTF or the presence of SGMA as Omicron SARS-CoV-2 variant positive could have led to elide of Omicron SARS-CoV-2 variant cases of BA.2 lineage, which does not have the property of S gene dropout. The data regarding the pre-existence of comorbid conditions were not included in the current study, which might have resulted in interference in the estimation of the severity of the disease. We were not able to perform genomic sequencing to identify the different variants.

## Conclusions

Even though the clinical course of the Omicron SARS-CoV-2 variant is predominantly milder with fewer hospitalization, the meteoric rise of cases can furor the healthcare system and policymakers and can create havoc, demanding the good preparedness of the medical management system to tackle all the predicaments. Furthermore, vaccine coverage should be kept at the apex level of action and stewardship program as a safeguard in all groups, irrespective of the variant type.
